# M2-polarized macrophages in keratocystic odontogenic tumor: relation to tumor angiogenesis

**DOI:** 10.1038/srep15586

**Published:** 2015-10-28

**Authors:** Wen-Qun Zhong, Gang Chen, Wei Zhang, Xue-Peng Xiong, Yi Zhao, Bing Liu, Yi-Fang Zhao

**Affiliations:** 1 State Key Laboratory Breeding Base of Basic Science of Stomatology (Hubei-MOST) and Key Laboratory of Oral Biomedicine Ministry of Education, School and Hospital of Stomatology, Wuhan University, Wuhan, China; 2 Department of Oral and Maxillofacial Surgery, School and Hospital of Stomatology, Wuhan University, Wuhan, China; 3 Department of Prosthodontics, School & Hospital of Stomatology, Wuhan University, Wuhan, China

## Abstract

The purpose of this study was to evaluate the presence of M2-polarized macrophages and their relationships to angiogenesis in keratocystic odontogenic tumor (KCOT). M2-polarized macrophages were detected in KCOT samples by immunohistochemistry and immunofluorescence. Meanwhile, microvessel density measured with antibody against CD31 was closely correlated with the presence of M2-polarized macrophages. In addition, macrophage colony-stimulating factor (M-CSF) significantly contributed to the activation of M2-polarized macrophages. Moreover, the results of *in vitro* wound healing, cell migration and tube formation assays further revealed the pro-angiogenic function of M2-polarized macrophage-like cells. This function might be associated with secretion of angiogenic cytokines, such as vascular endothelial growth factor (VEGF), transforming growth factor-β (TGF-β) and matrix metalloprotein-9 (MMP-9). This study demonstrates for the first time that M2-polarized macrophages are prevalent in KCOT, and their presence is dependent on M-CSF expression. More importantly, these tumor-supportive cells can also promote tumor angiogenesis by secreting angiogenic cytokines.

Keratocystic odontogenic tumor (KCOT) is one of the most common tumors arising from odontogenic epithelium^1^. KCOT is a benign but rapidly growing aggressive odontogenic neoplasm associated with nevoid basal cell carcinoma syndrome^2^. Although conventional studies on KCOT focused on epithelial lining, recent studies indicated that the stromal components of KCOT might promote vital processes, including tumor growth, invasion, and angiogenesis^3^,^4^. Hence, the main cells in the KCOT stroma and their precise roles must be elucidated.

Macrophages are the major cellular population in the tumor stroma and feature remarkable diversity and plasticity^5^,^6^. Macrophages acquire two different phenotypes, which are dependent on various signals in the tumor microenvironment. Classical M1-polarized macrophages, which are activated by microbial products and interferon-γ, highly express CD68 and human leukocyte antigen (HLA)-DR^7^,^8^. M2-polarized macrophages can exhibit pro-inflammatory and anti-tumor activities^5^, while alternative M2-polarized macrophages (activated by interleukin-4 [IL-4] and interleukin-13 [IL-13]) facilitate anti-inflammation and tumor progression and thus are referred as tumor-associated macrophages (TAMs)^6^. M2-polarized macrophages, which express high levels of CD68 and CD163, may promote tumor progression through immunosuppression, invasion, and angiogenesis^5^,^9^. However, the function of macrophages in KCOT remains largely unknown.

In this study, we reported, for the first time, that M2-polarized macrophages were present and contributed to angiogenesis in KCOT. As previous studies have confirmed that tumor angiogenesis greatly contributed to the growth potential and locally aggressive behavior of KCOT^10^,^11^,^12^,^13^, our findings provide novel insights into the pathology of KCOT and may facilitate the development of new treatment approaches.

## Results

### Infiltration of M2-polarized macrophages in KCOT

**Figure 1:**
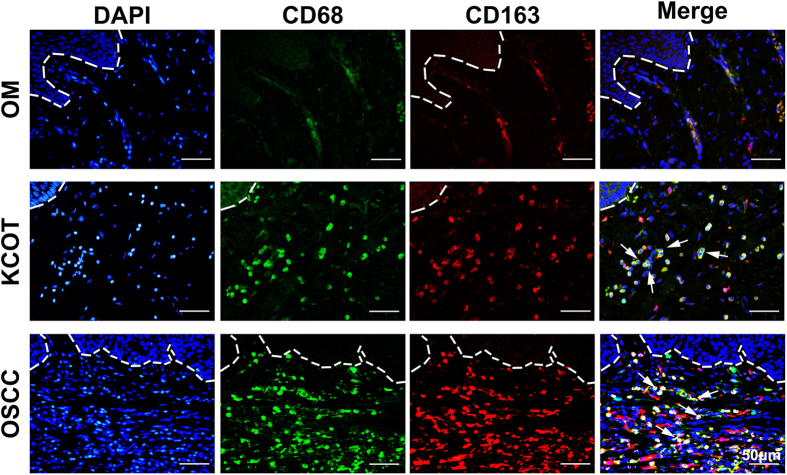
Detection of M2-polarized macrophages in KCOT using immunofluorescence. Double-labeling immunofluorescence for CD68 and CD163 in OM, KCOT and OSCC samples. The arrowheads indicate the CD68^+^/CD163^+^M2- polarized macrophages cells.

We tested the presence and distribution of M2-polarized macrophages in KCOT samples via immunofluorescence for CD68 and CD163 while M1-polarized macrophages were detected via immunostaining for CD68 and HLA-DR. Six oral mucosa (OM) and six oral squamous cell carcinoma (OSCC) samples were used as negative and positive controls, respectively. As shown in Supplementary Figure 1 and Fig. 1, CD68^+^/HLA-DR^+^ (M1-polarized macrophages) cells and CD68^+^/CD163^+^ (M2-polarized macrophages) cells were not detected in OM samples but were observed in OSCC samples. Our data showed that either M2-polarized or M1-polarized macrophages were present in 32 of the 34 KCOT samples As shown in Fig. 1, CD68^+^ and CD68^+^/CD163^+^ cells were observed in the stromal component of KCOT tissues, mainly located around the perivascular-like region and in the invasive front. Immunohistochemical analyses for CD68, HLA-DR and CD163 were also performed (Supplementary Figure 2). The ratios of CD68^+^, CD68^+^/HLA-DR^+^ and CD68^+^/CD163^+^ cells in the total cells varied among the KCOT tissue samples. The results of manual counting in five random sections revealed the following: (a) the ratio of CD68^+^ cells in the total cells ranged from 0% to 62.18%, with an average value of 29.91 ± 17.36%; (b) the ratio of CD68^+^/HLA-DR^+^ in the total cells ranged from 0% to 44.95%, with an average value of 12.31 ± 10.08%; and (c) the ratio of CD68^+^/CD163^+^ in the total cells ranged from 0% to 34.58%,with an average value of 16.04 ± 11.01%.

### M2-polarized macrophages were positively correlated with angiogenesis in KCOT samples

We evaluated the correlation of M2-polarized macrophages with tumor angiogenesis to explore their roles in KCOT. Previous evidences demonstrated that microvessel density (MVD) could reflect the angiogenesis of tumor tissues^14^,^15^. Thus, we initially determined the level of MVD by using anti-CD31 antibody. As shown in Supplementary Figure 3, CD31-positive vessels were mainly distributed in the connective tissues adjacent to the cystic epithelium lining and invasive front, similar to the description of previous publications^11^,^12^,^13^. MVD was higher in KCOT than that in OM (*P* < 0.05) but less than that in OSCC (*P* < 0.01). Interestingly, the MVD showed positive correlation with cellular proliferation marker (PCNA) and cellular invasive marker (MMP-9), respectively, which is in line with previous studies^10^,^13^ (Supplementary Figure 3C–E).

**Figure 2:**
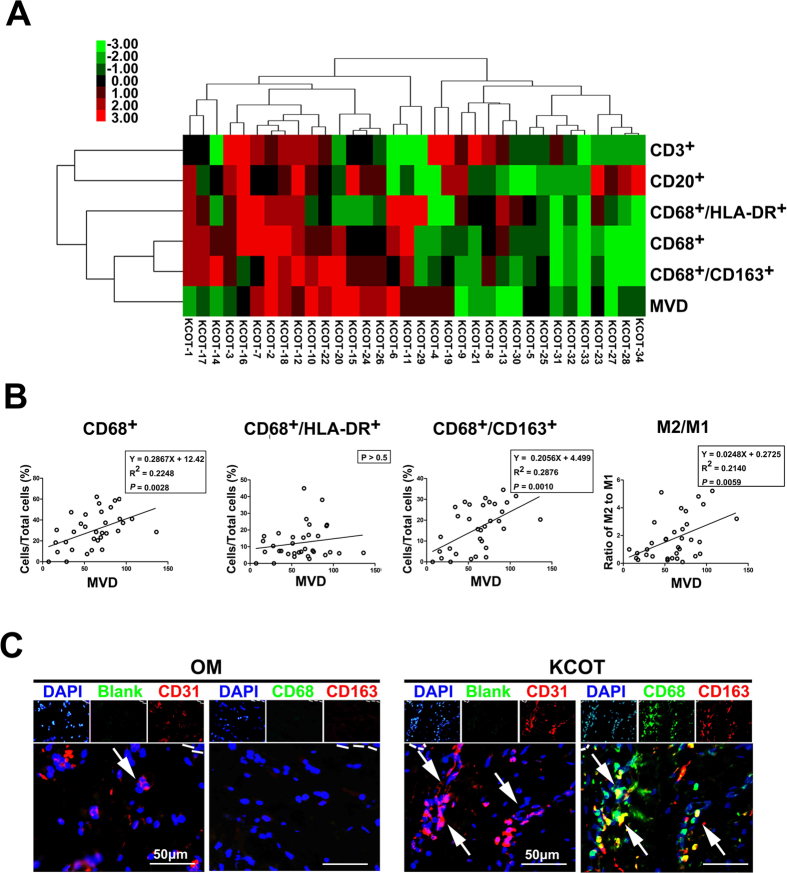
Correlation between M2-polarized macrophages and tumor angiogenesis. (**A**) Hierarchical clustering analysis for tested markers in KCOT samples. (**B**) Spearman rank correlation test and linear regression were performed to determine the correlation of MVD with CD68^+^, CD68^+^/HLA-DR^+^ and CD68^+^/CD163^+^, respectively. (**C**) Double-labeling immunofluorescence analyses for CD31 and CD68^+^/CD163^+^ were used to determine the colocalization of vessels and M2-polarized macrophages in the serial sections of KCOT samples. The proximity of vessels with M2-polarized macrophages could not been detected in OM tissue samples.

Not only macrophages, but CD3^+^ T cells and CD20^+^ B cells were also observed in the KCOT stroma (Supplementary Figure 4). To determine the correlation of these inflammatory cells with MVD, we performed a hierarchical clustering analysis. The results were visualized with a heat-map (Fig. 2A). In the heat-map, the length and subdivision of the branches directly reflected the correlation between the tested markers (top) and samples (left). In the whole group, CD68^+^, CD68^+^/HLA-DR^+^ and CD68^+^/CD163^+^ cells clustered closely with one another. These macrophages showed a closer correlation with MVD than both CD3^+^ T cells and CD20^+^ B cells. To verify the potential significance of these inflammatory cells in angiogenesis in KCOT, we investigated the correlations between MVD and the ratio of CD3^+^, CD20^+^, CD68^+^, CD68^+^/HLA-DR^+^ and CD68^+^/CD163^+^ cells in the total KCOT cells by using Spearman’s rank test. The MVD of KCOT was positively correlated with CD68^+^/CD163^+^ cells but not with CD68^+^/HLA-DR^+^ cells (Fig. 2B). MVD was also correlated with the ratio of CD68^+^ macrophage cells (Fig. 2B), but not CD3^+^ T cells, and CD20^+^ B cells in the total KCOT cells (Supplementary Figure 4). In addition, to provide a more definitive analysis, the correlation between MVD and the ratio of CD68^+^/CD163^+^ to CD68^+^/HLA-DR^+^ was performed. The result revealed that the ratio of CD68^+^/CD163^+^ to CD68^+^/HLA-DR^+^ was significant correlation with MVD in KCOT samples (Fig. 2B).

Double-labeling immunofluorescence analyses were then performed in the serial sections of KCOT tissue samples to validate the correlation between M2-polarized macrophages and tumor angiogenesis. As shown in Fig. 2C, CD68^+^/CD163^+^ M2-polarized macrophages were located around CD31-positive vessels. Collectively, these results implied that CD68^+^/CD163^+^ M2-polarized macrophages contributed to enhanced angiogenesis in KCOT.

### Homogenate supernatant of enucleation-treated KCOT tissues promoted the production of M2-polarized macrophages-like cells

**Figure 3:**
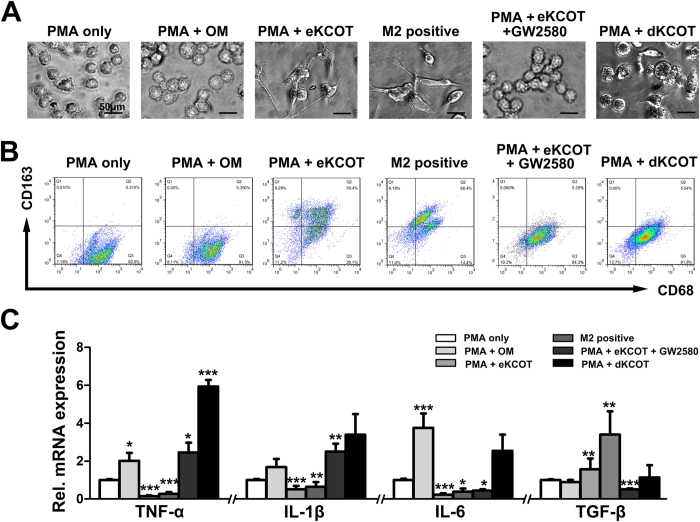
Induction of M2-polarized macrophages-like cells by homogenate supernatant of KCOT tissue. (**A**) Morphological changes of macrophage phenotypes. PMA only-treated, PMA + OM and PMA + dKCOT (secondary enucleation after decompression) homogenate supernatant-treated THP-1 cells appeared like a more round morphology, while M2-polarized macrophages induced by IL-4/IL-13 in positive control group turned out to be a more elongated shape. The morphology of most THP-1 cells treated with PMA + eKCOT (KCOT treated with enucleation alone) extract were also rather elongated, spindle-like shapes as the M2-polarized macrophages. (**B**) Expression of classical M2-polarized macrophages surface markers was evaluated by flow cytometry. The THP-1 cells showed a CD68^+^/CD163^+^ phenotype after the addition of PMA and eKCOT homogenate supernatant as the M2-polarized macrophages, but distinct from the PMA only, PMA + OM or PMA + dKCOT homogenate supernatant-treated macrophages. (**C**) The classical cytokine profiles of these macrophage populations were detected by real-time PCR. PMA + eKCOT homogenate supernatant-treated THP-1 displayed a low TNF-α, IL-1β, IL-6 and high TGF-β status as M2 type macrophages. Data are shown as the mean ± SEM. *P < 0.05, **P < 0.01, and ***P < 0.001 versus PMA only group.

To determine the effect of tumor homogenate supernatant on PMA-treated TPH-1 cells, we treated the cells with 25 ng/ml PMA for the first 12 hours and added with or without fresh OM or enucleation alone-treated KCOT (eKCOT) tissue supernatant for another 36 hours. Cells were also treated with IL-4/IL-13 to set as the positive controls. As shown in Fig. 3A, the treated THP-1 cells underwent morphological changes characterized by increased size and improved adherence, implying their differentiation from monocytes into macrophages. THP-1 cells treated with PMA only or PMA + OM homogenate supernatant displayed round morphology, whereas M2-polarized macrophages induced by IL-4/IL-13 were elongated. The morphology of most THP-1 cells cultured with PMA + eKCOT homogenate supernatant was also elongated and spindle like, which was similar to that of M2-polarized macrophages.

Flow cytometry was then performed to investigate the expression of surface markers for these cells. THP-1 cells treated with PMA only and PMA + OM homogenate supernatant demonstrated CD68^+^/CD163^−^ phenotype, whereas THP-1 cells treated with PMA + eKCOT homogenate supernatant showed CD68^+^/CD163^+^ phenotype (Fig. 3B).

We further compared the classical cytokine profiles of THP-1 cells treated with PMA only, PMA + OM and PMA + eKCOT homogenate supernatant. The cells treated with PMA only or PMA + OM homogenate supernatant shared similar profiles, including high TNF-α, IL-1β, and IL-6 levels and low TGF-β level. By contrast, the cells treated with PMA + eKCOT homogenate supernatant were similar to M2-polarized macrophages in terms of low TNF-α, IL-1β, and IL-6 levels and high TGF-β level (Fig. 3C). These results suggested that eKCOT homogenate supernatant could induce production of M2-polarized macrophages-like cells (M2L-macrophages) from PMA-treated THP-1macrophages.

### Macrophage colony-stimulating factor (M-CSF) was positively associated with the presence of M2-polarized macrophages in KCOT

**Figure 4:**
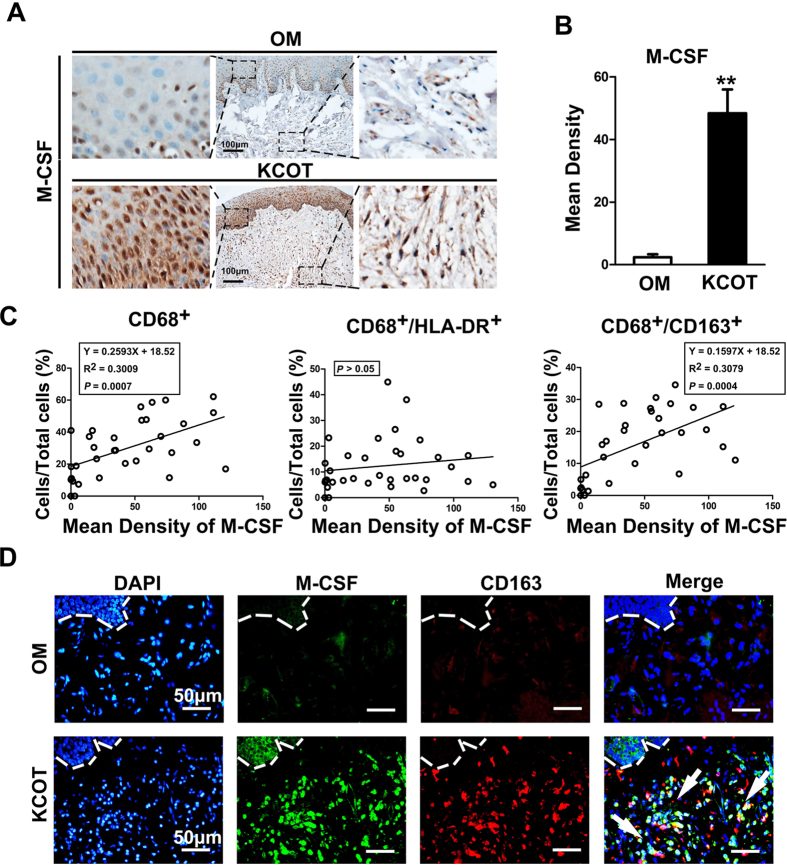
The correlation of M-CSF with the number of M2-polarized macrophages in KCOT samples **(A)** Increased expression of M-CSF in both epithelial and stromal sides of KCOT samples was found compared with this OM. **(B)** Quantification of M-CSF expression levels in both OM and KCOT samples. **(C)** The number of CD68^+^, CD68^+^/CD163^+^, but CD68^+^/HLA-DR^+^ macrophages showed a closely positive correlation with the expression of M-CSF. (C) Double-labeling immunofluorescence for M-CSF and CD163 in both of OM and KCOT samples. The white arrowheads indicated the M-CSF^+^ cells. Data are shown as the mean ± SEM. **P < 0.01versus OM group.

Numerous studies have shown that M-CSF (also known as colony-stimulating factor-1[CSF-1]) played a key role in induction and activation of M2-polarized macrophages in various tumors^16^,^17^. In this regard, we evaluated the expression levels of M-CSF in OM and KCOT tissue samples via immunohistochemistry. In line with our hypothesis, the expression level of M-CSF in KCOT samples was higher than that in OM samples (Fig. 4A,B). The results of Spearman rank’s test revealed a close correlation between the expression level of M-CSF and the ratio of CD68^+^ or CD68^+^/CD163^+^ cells in the total cells in KCOT (Fig. 4C). Moreover, as most CD163^+^ cells co-expressed CD68 in KCOT, we used CD163 to locate M2-polarized macrophages in subsequent experiments. Double-labeling immunofluorescence analyses for M-CSF and CD163 were further conducted on KCOT samples. As shown in Fig. 4D, CD163^+^ cells were widely co-expressed with M-CSF or closely surrounded by M-CSF^+^ cells. This observation implied that M-CSF could promote the phenotypic skewing of macrophages into the M2-polarized response through autocrine and paracrine pathways.

### GW2580 significantly inhibited the M2-polarized response of macrophages cultured in conditioned medium (CM) added with eKCOT tissue supernatant

**Figure 5:**
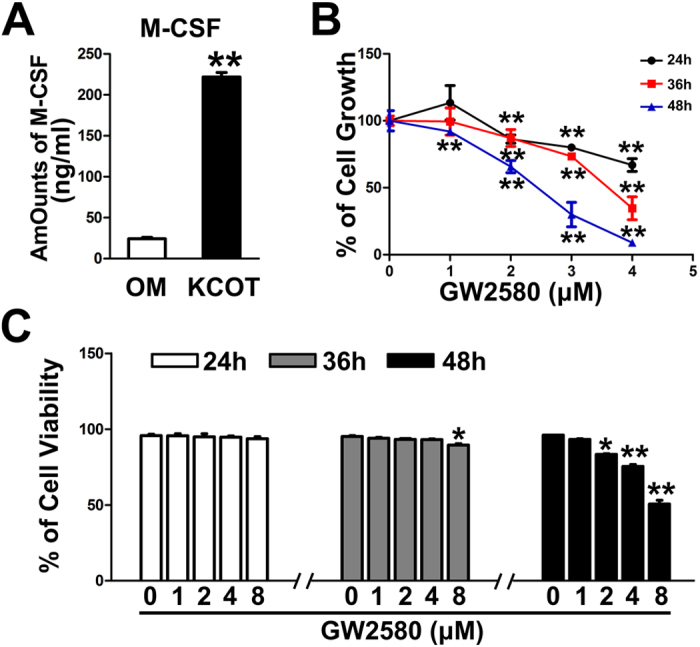
Evaluation of the concentration of M-CSF in tissue homogenate supernatant of OM and KCOT and the effect of GW2580 on PMA-treated macrophages’ growth and viability. (**A**) ELISA was performed to evaluate the concentration of M-CSF in tissue homogenate supernatant of OM and KCOT. (**B**) PMA-treated macrophages growth was determined by the MTT assay after treatment with various concentrations of GW2580 for 24, 36, and 48 h. The results were demonstrated as a percentage of the control group. (**C**) PMA-treated macrophages viability was measured by using the Vi-CELL cell viability analyzer after treatment with various concentrations of GW2580 for 24, 36, and 48 h. The results were demonstrated as the percentages of the control group. Data are shown as the mean ± SEM. *P < 0.05, and **P < 0.01versus the control group.

M-CSF content in homogenate supernatant was analyzed with an enzyme-linked immunosorbent assay (ELISA) kit to determine the role of M-CSF during polarization of THP-1 cells. As expected, the results showed that the amount of M-CSF in the eKCOT homogenate supernatant (222.02 ± 13.40 ng/ml) was significantly higher than that in OM homogenate supernatant (24.34 ± 4.15 ng/ml) (Fig. 5A). GW2580, a specific inhibitor of CSF-1R^18^,^19^, was used to block the action of M-CSF *in vitro*. We determined the growth of PMA-treated THP-1 cells in response to GW2580 treatment (12 hours after PMA) by using MTT. As shown in Fig. 5B, treatment with increased GW2580 concentrations substantially inhibited the growth of PMA-treated THP-1 cells. However, the results of cell viability assays demonstrated that treatment with different GW2580 concentrations (0–4 μM) for 36 hours did not affect the viability of PMA-treated THP-1 cells (Fig. 5C). Therefore, we set the 36-hour treatment period with GW2580 (0, 1, 2 and 4 μM) as the nontoxic condition for subsequent studies. THP-1 cells were then cultured in eKCOT homogenate supernatant with or without GW2580. As shown in Fig. 3, the morphological changes, the results of flow cytometry and cytokine profile analyses collectively revealed that GW2580 (4 μM) inhibited M2 polarization of PMA-treated THP-1 cells.

### M2L-macrophages facilitated angiogenesis *in vitro*

**Figure 6:**
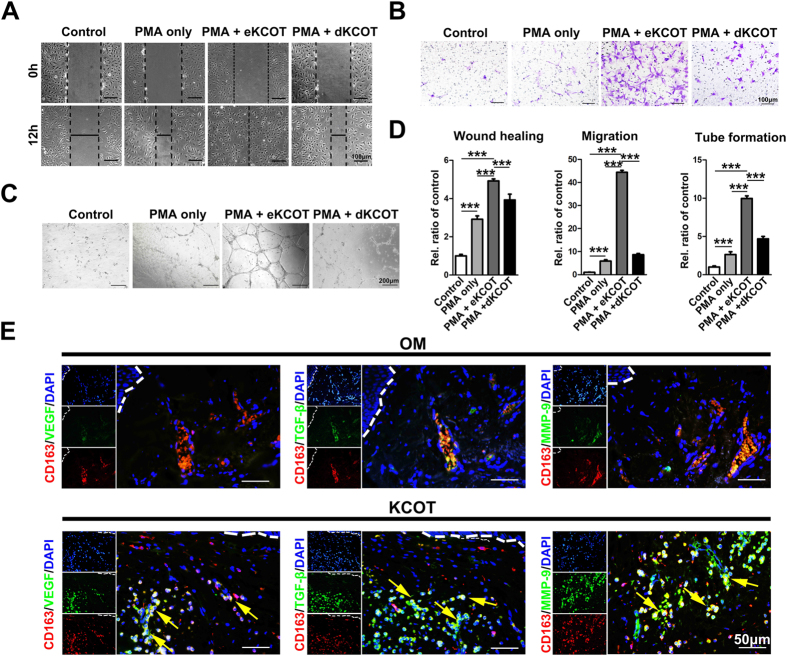
M2-polarized macrophages-like cells (M2L-macrophages) enhance HUVECs migration, tube formation. Meanwhile, M2-polarized macrophages promote tumor angiogenesis by secreting pro-angiogenic cytokines. (**A**,**B**) The enhanced cell migration was measured by wound healing and transwell system assays. (**C**) The increased formation of capillary-like structures was determined by tube formation assays. (**D**) Quantification of migration and capillary-like structures in wound healing, transwell migration and tube formation assays. The results were represented as relative ratios to the control group. (**E**) Double-labeling immunofluorescence staining for CD163 and VEGF, MM-9, TGF-β, respectively, both in OM and KCOT tissue samples. Data are shown as the mean ± SEM. NS, *P* ≥ 0.05, *P < 0.05, and **P < 0.01versus the control group.

As previous studies demonstrated that endothelial cells must undergo migration and tube formation during angiogenesis^20^,^21^, we cultured human umbilical vein endothelial cells (HUVECs) in different CMs to determine whether M2L-macrophages promote angiogenesis. As shown in Fig. 6A,B,D, the results of wound healing and transwell assays indicated that co-culture with CM from PMA + eKCOT tissue supernatant-treated THP-1 cells (PMA + eKCOT-CM) for 12 hours enhanced the migration of endothelial cells compared with these cultured in CM from THP-1 cells treated with PMA only (PMA only-CM). The results form tube formation assay further showed that exposure to PMA + eKCOT-CM could induce tube formation, whereas exposure to PMA only-CM weakened HUVEC tube formation (Fig. 6C,D). Hence, these above results suggested that the M2L-macrophages cells were capable of promoting angiogenesis.

### M2-polarized macrophages promoted KCOT angiogenesis by secreting angiogenic cytokines

To further investigate the mechanism of M2-polarized macrophages in mediating angiogenesis in KCOT, we performed double-labeling immunofluorescence for CD163/vascular endothelial growth factor (VEGF), CD163/transforming growth factor-β (TGF-β), and CD163/matrix metalloprotein-9 (MMP-9) on KCOT samples. Previous studies have demonstrated that M2-polarized macrophages could deliver angiogenic molecules, such as VEGF, TGF-β, and MMPs^22^,^23^. Similarly, our data revealed the co-expression of CD163/VEGF, CD163/TGF-β and CD163/MMP-9 in KCOT samples. All co-expression relationships were distributed in the perivascular-like sites of KCOT (Fig. 6E). In addition, most of the strong fluorescence signals for these major angiogenic cytokines were synchronously located in CD163-positive cells in KCOT. These findings suggest that M2-positive macrophages may be a major source of angiogenic factors.

### Decompression down-regulated M2-polarized macrophages and angiogenesis in KCOT

**Figure 7:**
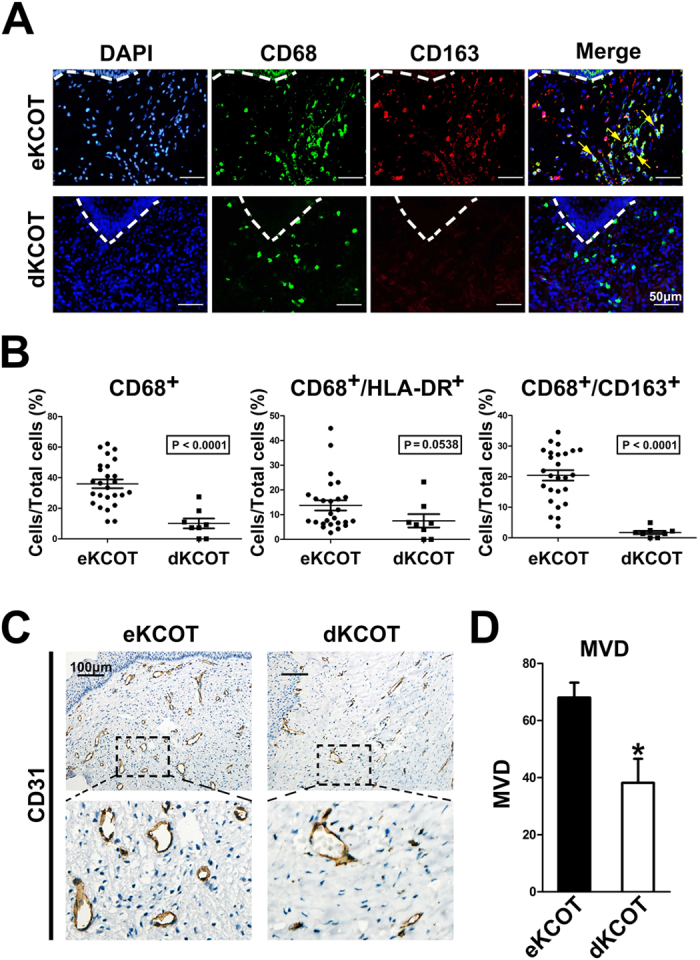
Detection of M2-polarised macrophages and MVD in eKCOT and dKCOT. (**A**) Double-labeling immunofluorescence for CD68 and CD163 in eKCOT and dKCOT samples. The arrowheads indicate the CD68^+^/CD163^+^ M2-polarized macrophages cells. (**B**) Down-regulation of CD68^+^ macrophages and CD68^+^/CD163^+^ M2-polarized macrophages in dKCOT were observed when compared with these in eKCOT. (**C**) Down-regulation of MVD in dKCOT was found when compared with eKCOT. (**D**) Quantification of MVD level in both eKCOT and dKCOT. Data are shown as the mean ± SEM. *P < 0.05 versus the eKCOT group.

For clinical significance, we compared presence of macrophages in eKCOT and dKCOT (KCOT treated by secondary enucleation after decompression) samples through double-labeling immunofluorescence for CD68 and CD163. In line with the preceding data, M2-polarized macrophages were abundantly detected in eKCOT. A weak immunoreaction for CD68^+^ cells was observed in dKCOT, whereas the immunoreaction for CD163^+^ and CD68^+^/CD163^+^ cells was rarely observed in dKCOT (Fig. 7A). The results of Student’s *t*-test further revealed that the ratios of CD68^+^ and CD68^+^/CD163^+^ cells in the total cells significantly decreased in dKCOT relative to those in eKCOT samples, which indicated that the presence of M2-polarized macrophages was significantly positive correlation with the treatment method, while the ratio of CD68^+^/HLA-DR^+^ cells in total cells did not significantly differ between the two groups (Fig. 7B). Consistently, the tumor homogenate supernatant of dKCOT failed to draw PMA-treated TPH-1 cells into M2-polarized macrophages according to Fig. 3. Meanwhile, *in vitro* studies also indicated that PMA + dKCOT-CM treated THP-1 cells lacked the capability to promote angiogenesis (Fig. 6). Thus, we explored the angiogenic phenotypes in both eKCOT and dKCOT samples through immunohistochemistry for CD31. As expected, the results revealed that the MVD was down-regulated after decompression, which indicated that the tumor angiogenesis was significant correlation with the treatment (Fig. 7C,D). Collectively, these results demonstrated that decompression in KCOT restrained the phenotypic skewing of macrophages into the M2-polarized types, and suppressed tumor angiogenesis.

## Discussion

M2-polarized macrophages, also known as TAMs, are primary cellular component of the tumor stroma and important in facilitating tumor maintenance and growth. M2-polarized macrophages exert their pro-tumor functions by enhancing immunosuppression, invasion, angiogenesis, and metastasis^5^,^24^. Nevertheless, the presence and potential roles of M2-polarized macrophages in KCOT, a benign odontogenic tumor with infiltrative behavior, remain largely unknown.

This study demonstrated for the first time the presence and distribution of M2-polarized macrophages as well as M1-polarized macrophages in the KCOT stroma. The number of M2-polarized macrophages was significantly correlated with MVD, a well-known marker for tumor vascularization^25^,^26^. Thus, we investigated the mechanisms underlying macrophage polarization and their potential relationships to angiogenesis in KCOT.

The phenotypic skewing of macrophages depends on an intricate crosstalk between tumor cells and their cellular and extracellular microenvironments^24^. Generally, the culture supernatant of tumor cells is often used for research *in vitro*, and addition of CM of various carcinoma cells is also utilized to determine macrophage polarization. This method can succinctly reflect the tumor microenvironment to some extent. However, these carcinoma cells differ from the neoplasm microenvironment *in vivo* because of the complex and mutual interactions between tumor cells and various stromal cells. In this regard, the supernatant of tumor tissue homogenate is regarded as an appropriate material to simulate the tumor microenvironment^27^. By contrast, researches aimed to explore the influence of the tumor microenvironment on tumor epithelial and stromal cells in KCOT have been hampered because of insufficient stabilized and recognized cell populations. Given this limitation, we prepared CM by adding KCOT homogenate supernatant to investigate the effect of the supernatant on macrophage polarization. As expected, the homogenate supernatant of eKCOT tissues induced M2-like polarization in PMA-treated macrophages. The number of M2-polarized macrophages was also significantly correlated with M-CSF expression in KCOT samples. This finding indicated the key role of M-CSF played in the M2-polarized response of macrophages. Moreover, the quantified amount of M-CSF in eKCOT homogenate supernatant was higher than that in normal OM. GW2580, a specific M-CSFR inhibitor, was used to block the effect of M-CSF to further investigate the underlying mechanisms. The results demonstrated that GW2580 could effectively inhibit the M2-polarized response of PMA-treated macrophages. Therefore, KCOT tissue homogenate supernatant can induce the production of M2-polarized macrophages in an M-CSF-dependent manner.

To provide evidence for our hypothesis that M2-polarized macrophages might play a key role in angiogenesis in KCOT, we collected CMs from M2L-macrophage cells and macrophages treated with PMA only. Moreover, the effect of CMs on the migration and tube formation of HUVECs was measured *in vitro* through wound healing, cell migration and tube formation assays. Data showed that the CM from M2L-macrophage cells could significantly enhance the motility and tube formation of HUVECs compared with that from macrophages treated with PMA only. These results confirm that M2-polarized macrophages may considerably affect angiogenesis in KCOT.

Basically, the new vessel formation is necessary to supply nutrients and oxygen, which are indispensable for survival and rapid growth of tumor cells. Numerous studies have revealed that M2-polarized macrophages played a central role in controlling tumor angiogenesis^23^. According to these studies, TAMs usually served as a major source of angiogenic factors and could release various pro-angiogenic factors^22^,^23^. In the present study, M2-polarized macrophages co-expressed VEGF, MMP-9, and TGF-β. Besides, the synchronous distribution of these angiogenic cytokines and M2-positive macrophages in KCOT further revealed that M2-polarized macrophages might function as a primary source of angiogenic molecules. Collectively, these results demonstrate that M2-polarized macrophages can promote angiogenesis by secreting various cytokines in KCOT.

Previous studies, including our previous work, showed that decompression was an effective treatment strategy for KCOT, particularly for those with large sizes^28^. Nevertheless, the exact pathological process of KCOT after decompression, particularly in connective tissues, remains unknown. For clinical significance, we explored the changes in macrophages and MVD in KCOT after decompression. The results showed that CD68^+^ macrophages, CD68^+^/CD163^+^ M2-polarized macrophages and the MVD decreased in KCOT after decompression. In addition, decompression also reduced the ability of KCOT homogenate supernatant to promote the production of M2-polarized macrophages, and decompression could also suppress tumor angiogenesis. Therefore, decompression can lead to anti-tumor transformation of the intrinsic pro-tumor connective tissues, which can be a novel evidence supporting the use of decompression for conservative management of KCOT.

In summary, this study revealed, for the first time, the presence and distribution of M2-polarized macrophages in KCOT and their key roles in angiogenesis in KCOT. As specific intervention strategies targeting TAMs have emerged as a desirable perspective and have made some considerably progresses, they can also be further developed into KCOT. However, future studies must investigate the origin and precise differentiation processes of macrophages in KCOT.

## Methods

### Samples, immunohistochemistry, double-labeling immunofluorescence

Thirty-four KCOT samples, including 26 eKCOT and 8 dKCOT, 6 normal OM samples, and 6 OSCC samples were collected at the Hospital of Stomatology, Wuhan University. This study was approved by the review board from the ethics committee of the Hospital of Stomatology, Wuhan University. The procedures were carried out according to the National Institutes of Health guidelines regarding the use of clinical tissues. Meanwhile, written informed consent for this study was obtained from all the participants. All samples were fixed in buffered 4% paraformaldehyde and embedded in paraffin. Anti-human CD68 monoclonal antibodies were used to recognize macrophages. CD68, CD163 double immunohistochemical staining was performed to identify M2-polarized macrophages. On the other hand, CD68, HLA-DR double immunohistochemical staining was carried out to detect M1-polarized macrophages. MVD was determined by counting the number of vessels. KCOT diagnosis, immunohistochemistry, double-labeling immunofluorescence as described previously^9^,^29^,^30^.

### Preparation of tumor tissue homogenate

Tumor homogenate was prepared according to previously reported procedures^27^,^31^. Ten fresh eKCOT and seven dKCOT tissues were collected and washed thoroughly. Each KCOT tissue sample (100 mg) was immediately placed in 5 ml of serum-free RPMI 1640 medium and homogenized in a glass homogenizer. The supernatant was centrifuged at 10,000 *g* for 1 hour. Cell-free tissue supernatant was then collected and stored frozen at −80 °C for further use.

### Cell culture

THP-1 cells were cultured in RPMI 1640 medium supplemented with 10% FBS, 100 U/mlpenicillin, and 100 ng/ml streptomycin^32^. HUVECs were isolated from human umbilical cord veins as previously described^20^ and then cultured in endothelial basal medium supplemented with 20% FBS, SingleQuot (Bio Science), 100 U/ml penicillin, and 100 ng/ml streptomycin. The protocols for generating or inhibiting polarized THP-1 macrophages were described in Supplementary Materials and Methods.

### Real-time quantitative PCR

Basing on previously described procedures^33^, we isolated total RNA, synthesized cDNA and conducted real-time qPCR. GAPDH was selected as the internal control in our experiments. The primer nucleotide sequences for PCR were listed in Supplementary Materials and Methods.

### Preparation of CMs

After THP-1 cells were treated with PMA in the absence or presence of tissue supernatants for 48 hours, the mediums were changed and added with serum-free PRMI 1640. After 48 hours, CMs were collected and centrifuged at 500 *g* for 20 minutes and stored at −20 °C for further use. These CMs were named as follows: PMA only-CM (from THP-1 cells treated with PMA only), PMA + eKCOT-CM (from THP-1 cells treated with the PMA and eKCOT tissue supernatant) and PMA + dKCOT-CM (from THP-1 cells treated with the PMA and dKCOT tissue supernatant)

### Wound healing, migration and tube formation assays

Wound healing, migration and tube formation assays using HUVECs were performed as previously described^21^,^34^ in Supplementary Materials and Methods.

### Flow cytometry

After treatment with PMA in the absence or presence of tissue supernatant, THP-1 cells (1 × 10^6^) were collected for flow cytometric analyses through BD fluorescent-activated cell sorter system (Calibur, CO) to detect surface markers according to our previous procedures^35^,^36^. The results were analyzed with FlowJo 7.6.1 software (Tree Star, Inc., OR). The cells were surface-stained with FITC-CD68 and PE-CD163 according to the manufacturer’s instructions.

### ELISA

Human M-CSF level in KCOT tissue supernatant was measured through ELISA as previous steps^27^,^31^. An ELISA kit (DoBi, China) for human M-CSF was used according to the manufacturer’s instructions.

### Statistical analysis

To evaluate the ratio of different kinds of macrophages, 5 representative high-power fields (×400 magnification) of each specimen were randomly selected and the total number of cells counted by two blind researchers. The ratios for the positive cells were calculated by the formula as our former description^9^: positive ratio = positive cells number/total cells number. All data were presented as mean ± SEM of three independent experiments. One-way analysis of variance, Student–Newman–Keuls, and Spearman’s rank correlation test were performed for statistical analysis. *P* < 0.05 was considered statistically significant.

## Additional Information

**How to cite this article**: Zhong, W.-Q. *et al.* M2-polarized macrophages in keratocystic odontogenic tumor: relation to tumor angiogenesis. *Sci. Rep.* **5**, 15586; doi: 10.1038/srep15586 (2015).

## Supplementary Material

Supplementary Information
