# Supplementary material for: M2-polarized macrophages in keratocystic odontogenic tumor: relation to tumor angiogenesis
Source: Sci Rep. 2015 Oct 28;5:15586. doi: 10.1038/srep15586 (PMC4623606; doi:10.1038/srep15586)
Supplement: Supplementary Information [file srep15586-s1.pdf]

M2-polarized Macrophages in Keratocystic Odontogenic Tumor: Relation to Tumor Angiogenesis.

Wen-Qun Zhong[1], **Gang Chen**[1,2], **Wei Zhang**[1,2], **Xue-Peng Xiong**[1,2], **Yi Zhao**[1,3,] *, **Bing Liu** [1,2,] * &**Yi-Fang Zhao**[1,2]

[1]State Key Laboratory Breeding Base of Basic Science of Stomatology (Hubei-MOST) and Key Laboratory of Oral Biomedicine Ministry of Education, School and Hospital of Stomatology, Wuhan University, Wuhan, China; [2]Department of Oral and Maxillofacial Surgery, School and Hospital of Stomatology, Wuhan University, Wuhan, China; [3]Department of Prosthodontics, School & Hospital of Stomatology, Wuhan University, Wuhan, China.

Supplementary materials and methods

Expanded methods:

Immunohistochemistry

The immunohistochemical analysis was conducted as previously described previous[1-3.] The primary antibodies were as follows: proliferating cell nuclear antigen (PCNA) (1:400, Santa Cruz Biotechnology), B cell lymphoma 2 (Bcl-2) (1:200, Abcam), matrix metalloprotein-9 (MMP-9) (1:200, Santa Cruz Biotechnology), CD31 (1:200, Epitomics) and macrophage colony-stimulating factor (M-CSF) (1:200, Epitomics). For semi-quantitation, the images were analyzed with Image Pro software as previous procedures[1-3.]

Double-labeling immunofluorescence and immunohistochemistry.

The double-labeling immunofluorescence and immunohistochemical analysis were performed as previous description[1,4,5]. The primary antibodies used in these experiments were listed as follows: CD68 (Rabbit, Proteintech), CD163 (Mouse, Santa Cruz Biotechnology), CD31 (Rabbit, Epitomics), vascular endothelial growth factor (VEGF) (Rabbit, Cell Signaling Technology), transforming growth factor-β (TGF-β) (Rabbit, Proteintech) and matrix metalloprotein-9 (MMP-9) (Rabbit, Santa Cruz Biotechnology).

Cell culture.

According to previous description[6], THP-1 cells, obtained from ATCC, were cultured in RPMI 1640 medium supplemented with 10% FBS, 100 U/mL penicillin and 100 μg/mL streptomycin. To generate polarized THP-1 macrophages, one million THP-1 cells were seeded into six-well culture plates and treated with 25 ng/mL phorbol myristate acetate (PMA) for 48 hours. To obtain M2-polarized cells, THP-1 cells were treated with 25 ng/mL PMA for 48 hours and further polarized them with 20ng/ml IL-4 and 20ng/ml IL-13 for 36 hours, added 12 hours after PMA. M2-polarized macrophages-like (M2L-macrophages) were generated from THP-1 cells culture with 25 ng/mL PMA for 48 hours and eKCOT tumor homogenate for final 36 hours (30%, added 30 hours after PMA). To inhibit the influence of M-CSF on the differentiation of M2-polarized macrophages, GW2580, a specific M-CSFR inhibitor, was added to the medium as previous description[7,8]. HUVECs, isolated from human umbilical cord

veins by as our previously described[9], were cultured in endothelial basal medium (EBM) supplemented with 20% fetal bovine serum, SingleQuot (Bio Science), and 100 U/mL penicillin, and 100 ng/mL streptomycin. Passages 2–7 of these cells were used in this study. Cell densities were counted by the Vi-CELL cell viability analyser (Beckman Coulter) as our previous studies[9]. All experiments were repeated more than three times.

Cell viability assay.

One million 25ng/mL PMA-treated THP-1 cells were seeded in 6-well plates. After THP-1 cells were cultured in 25 ng/mL PMA for 12 h, they were treated with serum-free RPMI 1640 medium containing GW2580 (a specific antagonist for CSF-1R, Germany) at various concentrations for 24, 36, and 48 h. Then, total cells were analyzed by using a Vi-CELL cell viability analyzer, based on trypan blue exclusion (Beckman Coulter, USA) as our previous description[9,10]. All experiments were repeated three times at least.

MTT assay.

According to our previous steps[10], ten thousand 25ng/mL PMA-treated THP-1 cells plated onto 96-well plates for 12 h then, incubated for 24, 36, and 48 h with 100 ml of various concentrations of GW2580 in serum-free RPMI 1640 medium. Then, 10 μL of MTT solution (5 mg/ml) was added to each well and incubated for another 4 h at 37 ^{o}C. After that, the medium was changed, 150 μL DMSO was added to the wells, and

the absorbance was quantified at 490 nm by using a 96-well microplate reader (Bio-Tek). This experiment was repeated three times or more.

Real-time quantitative PCR.

As our previous description[11], total RNA was isolated, the cDNA was synthesized and real-time qPCR was performed. GAPDH was selected as an internal control in our experiment. The primer nucleotide sequences for PCR were listed as follows: TNF-α: 5’-TCTTCTCGAACCCCGAGTGA-3’ and 5’-CCTCTGATGGCACCACCAG-3’; IL-1β: 5’-TACGAATCTCCGACCACCACTACAG-3’ and 5’-TGGAGGTGGAGAG CTTTCAGTTCATATG-3’; IL-6: 5’-TACATCCTCGACGGCATCT-3’ and 5’-ACCAGGCAAGTCTCCTCAT-3’; TGF-β: 5’-GGGACTATCCACCTGCAAGA-3’ and 5’-CCTCCTTGGCGTAGTAGTCG-3’; GAPDH: 5’-CCATGTTCGTCATGGGT GTGAACCA-3’ and 5’-GCCAGTAGAGGCAGGGATGATGTTC-3’. Our experiments were repeated at least three times.

Endothelial cell wound healing assay.

HUVECs were seeded in six-well culture plates as our previous description [9]. When these cells grew to 90% confluence, a gap of constant width scraped with a micropipette tip was made in the center of the cell monolayers. The medium was changed and replaced by EBM with 20% FBS, 50% PMA only-CM, 50% PMA + eKCOT-CM or 50% PMA + dKCOT-CM. Twelve hours later, the cells were fixed and The distance of cell migration was calculated by Image J. Data were expressed as

the ratios of migration compared to control groups. This experiment was repeated at least three times.

Endothelial cell migration assay.

Endothelial cell migration was measured using a transwell Boyden chamber system (Becton-Dickinson). Briefly, HUVEC (5×10^5) cells were seeded into the upper chamber in 100μL of serum-deprived EBM, whereas EBM with 5 ng/ml VEGF, 50% PMA only-CM, 50% PMA + eKCOT-CM or 50%PMA + dKCOT-CM were added to the lower chambers as chemoattractants. After incubation at 37℃ for 12 hours, the cells in the upper surface of the chamber were thoroughly removed with a cotton swab, and the migrated cells were fixed with buffered 4% paraformaldehyde, followed by staining with crystal violet, and then photographed and quantified. The experiment was repeated at least three times.

Tube formation assay.

As our preceding steps[9], HUVECs (2×10^5 cells) in 500μL EBM with 10% FBS, 50% PMA only-CM, 50% PMA + eKCOT-CM or 50% PMA + dKCOT-CM were seeded onto forty-eight-well culture plates coated with BD Matrigel™ Matrix (Becton-Dickinson). After incubation for 24h at 37℃, cells were fixed, photographed under phase-contrast microscopy, and capillary-like structures were photographed at low-power magnification. Five random fields in each well were chosen to count and analyses. All experiments were repeated at least three times.

References

1. Zhong, W.Q., *et al.* Down-regulation of connexin43 and connexin32 in keratocystic odontogenic tumours: potential association with clinical features. *Histopathology* ***66, 798-807*** *(2015).* (2014).
2. West, X.Z., *et al.* Oxidative stress induces angiogenesis by activating TLR2 with novel endogenous ligands. *Nature* **467**, 972-976 (2010).
3. Chang, C.M., *et al.* Nerve sprouting and sympathetic hyperinnervation in a canine model of atrial fibrillation produced by prolonged right atrial pacing. *Circulation* **103**, 22-25 (2001).
4. Wang, F.Q., *et al.* M2-polarised macrophages in infantile haemangiomas: correlation with promoted angiogenesis. *J Clin Pathol* **66**, 1058-1064 (2013).
5. Jia, J., *et al.* Expression of allograft inflammatory factor-1 and CD68 in haemangioma: implication in the progression of haemangioma. *Br J Dermatol* **159**, 811-819 (2008).
6. Stewart, D.A., Yang, Y., Makowski, L. & Troester, M.A. Basal-like breast cancer cells induce phenotypic and genomic changes in macrophages. *Mol Cancer Res* **10**, 727-738 (2012).
7. Hume, D.A. & MacDonald, K.P. Therapeutic applications of macrophage colony-stimulating factor-1 (CSF-1) and antagonists of CSF-1 receptor (CSF-1R) signaling. *Blood* **119**, 1810-1820 (2012).
8. Mossadegh-Keller, N., *et al.* M-CSF instructs myeloid lineage fate in single haematopoietic stem cells. *Nature* **497**, 239-243 (2013).

9. Zhang, W., Chen, G., Ren, J.G. & Zhao, Y.F. Bleomycin induces endothelial mesenchymal transition through activation of mTOR pathway: a possible mechanism contributing to the sclerotherapy of venous malformations. *Br J Pharmacol* **170**, 1210-1220 (2013).

10. Liu, H., *et al.* Overexpression of macrophage migration inhibitory factor in adenoid cystic carcinoma: correlation with enhanced metastatic potential. *J Cancer Res Clin Oncol* **139**, 287-295 (2013).

11. Chen, G., *et al.* Disorganized vascular structures in sporadic venous malformations: a possible correlation with balancing effect between Tie2 and TGF-beta. *Sci Rep* **4**, 5457 (2014).

Supplementary figures and figure legends:

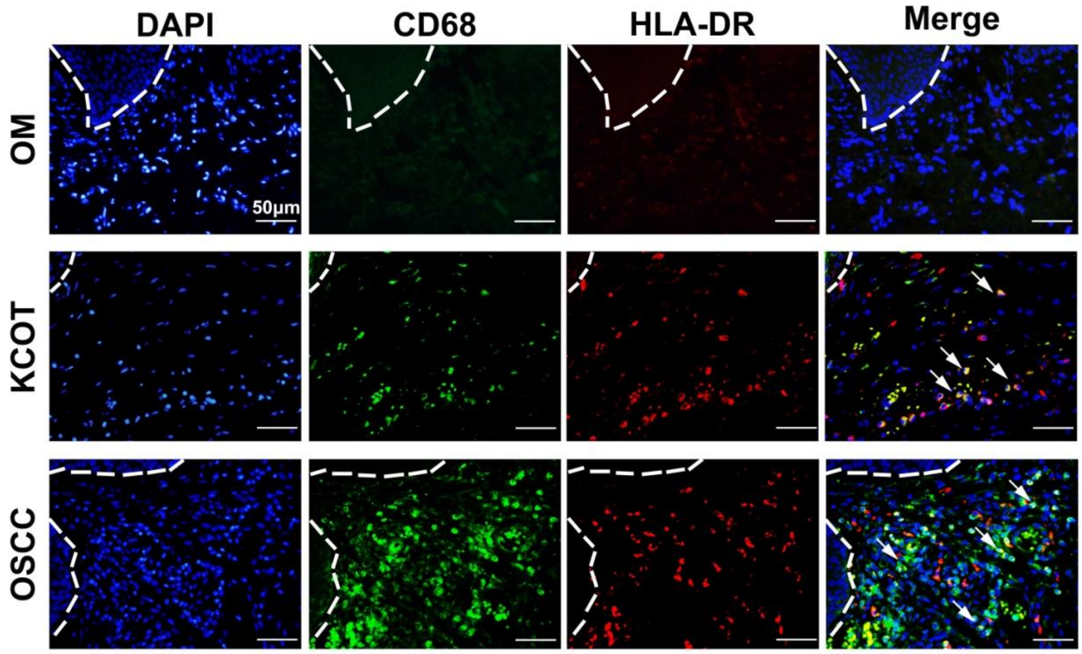

Figure 1S. Detection of M1-polarized macrophages in KCOT using immunofluorescence. Double-labeling immunofluorescence for CD68 and HLA-DR in OM, KCOT and OSCC samples. The arrowheads indicate the $CD68^{+}/HLA\text{-}DR^{-}$ M1- polarized macrophages cells.

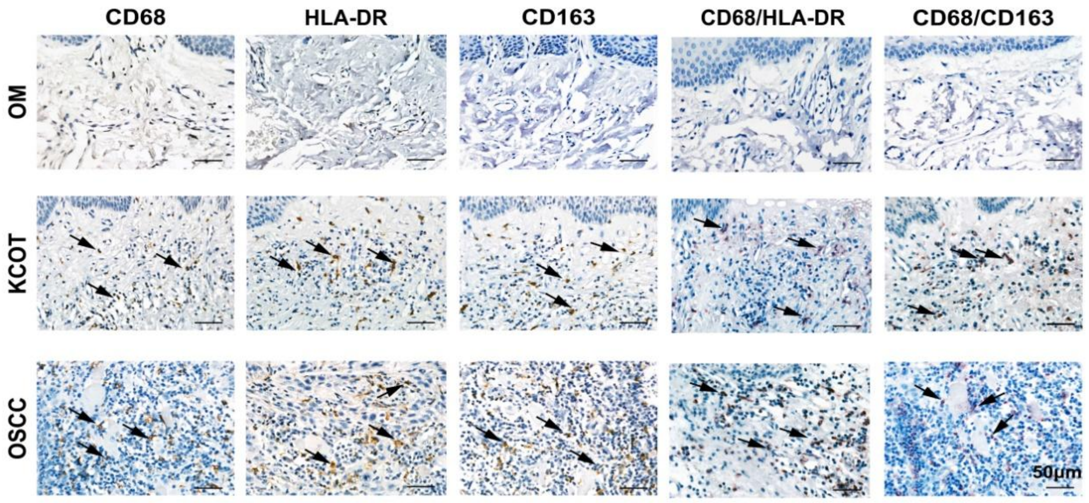

Figure 2S. Detection of M2-polarised macrophages in KCOT. Immunohistochemical staining of CD68, HLA-DR and CD163 was shown in the *first*, *second and third rows*. Meanwhile, double-labeling immunohistochemical staining for both CD68 (red staining of the cytoplasm)/HLA-DR (a dark purple cytoplasm staining) and CD68 (red staining of the cytoplasm)/CD163 (a dark purple cytoplasm staining) was also shown in the *fourth and fifth rows*, respectively. The arrows indicate the positive immunoreactivity for tested markers. Moreover, immunohistochemical staining for CD68 and HLA-DR as well as CD163 was performed in normal OM and OSCC samples as negative and positive control, respectively.

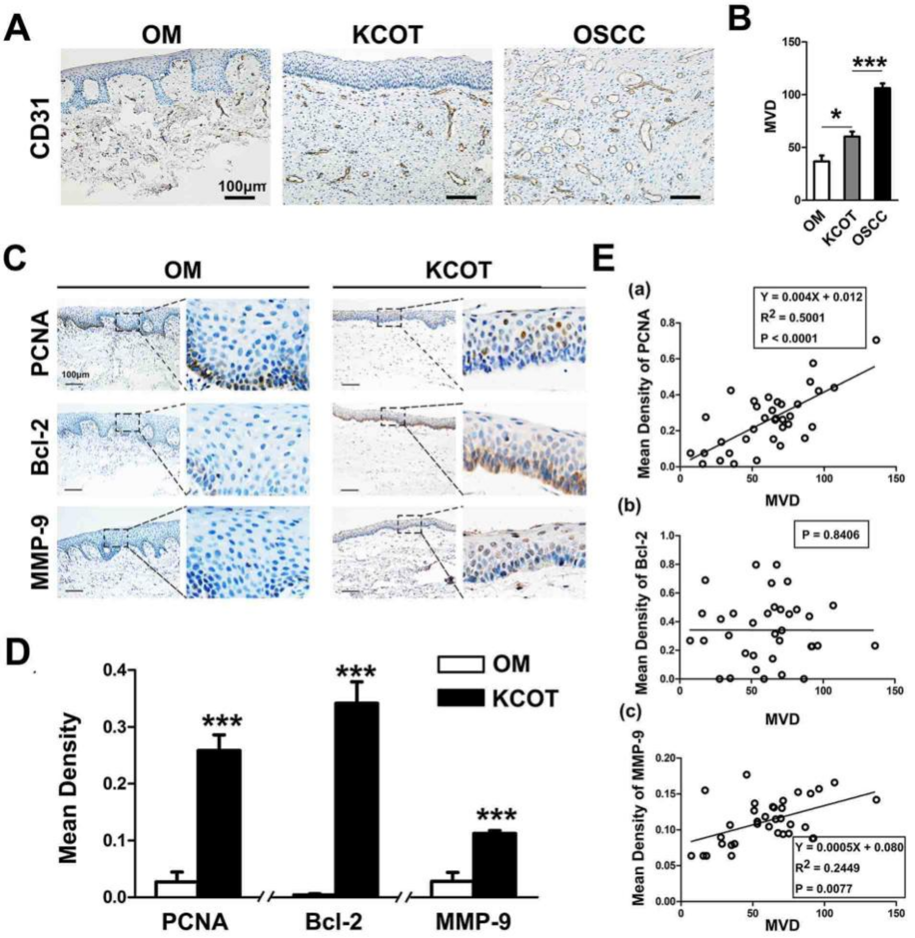

Figure 3S. Immunohistochemical staining with CD31 monoclonal antibody and the evaluation of MVD in OM and KCOT. **(A)** Staining of blood vessels with anti-CD31 antibody adjacent to epithelium. **(B)** Quantification of MVD levels in OM and KCOT. **(C)** Immunohistochemical staining of PCNA, Bcl-2 and MMP-9 in both of serial sections of OM and KCOT samples. **(D)** Increased expression of PCNA, Bcl-2 and MMP-9 in KCOT samples compare with these markers in OM samples. **(E)** Different from Bcl-2 (b), the expression levels of PCNA (a) and MMP-9 (c), showed a significantly positive correlation with the expression of MVD. Data are shown as the mean ± SEM. *, $p < 0.05$, and ***, $p < 0.001$versus the OM group.

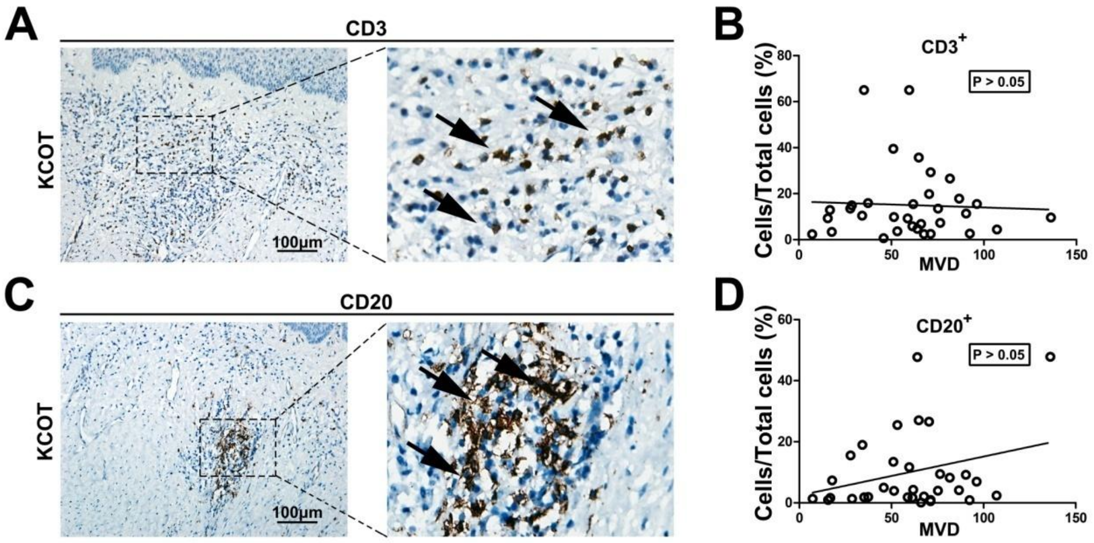

Figure 4S. Detection of $CD3^+$ T cells and $CD20^+$ B cells in KCOT by using immunohistochemistry and their correlation with tumor angiogenesis. **(A)** Immunohistochemical staining of CD3 in KCOT samples. **(B)** Correlation between $CD3^+$ T cells and MVD in KCOT. **(C)** Immunohistochemical staining of CD20 in KCOT samples. **(D)** Correlation between $CD20^+$ T cells and MVD in KCOT.
